# Psychosocial care network for children with autism spectrum disorder in brazil

**DOI:** 10.1192/j.eurpsy.2021.581

**Published:** 2021-08-13

**Authors:** D. Molini-Avejonas, V. Mandaj

**Affiliations:** Fonoaudiologia, Universidade de São Paulo, São Paulo, Brazil

**Keywords:** autism spectrum disorder, Psychosocial care network, Children, public health

## Abstract

**Introduction:**

The prevalence of ASD is 1 for every 59 children, an increase of 15%, referring to 2012. Studies supported the formulation of laws and guidance documents by the State in Brazil. Each location has found ways to meet their demands seeking to guarantee the needs of these users in public health services, whether through the Psychosocial Care Centers or the Rehabilitation Centers, healthcare clinics suggested by the Ministry of Health for the service to these users.

**Objectives:**

The general objective of this study is to characterize the psychosocial care network in Brazil, in order to verify whether these principles are considered in the line of care for ASD.

**Methods:**

A public service evaluation questionnaire was applied to analyze the users’ perception on the care network effectiveness.

**Results:**

There is lack of communication, matrix support or articulation and highlight that the construction of physical spaces does not always translate into an integrated intersectoral treatment. The articulation between the services and referrals involved could contribute to greater treatment control of this demand.

**Conclusions:**

Reflecting on the study, we consider that a network based on case severity care would be considerably more efficient, since the individual could use the services according to the demand present at the time. This proposal would create more humanized, personalized, assertive services, without financial waste, and would serve the system by guaranteeing the rights of integrality, universality and mainly of equity of these users within SUS.

